# Perforin enhances the granulysin-induced lysis of Listeria innocua in human dendritic cells

**DOI:** 10.1186/1471-2172-8-14

**Published:** 2007-08-16

**Authors:** Michael Walch, Sonja Latinovic-Golic, Ana Velic, Hanna Sundstrom, Claudia Dumrese, Carsten A Wagner, Peter Groscurth, Urs Ziegler

**Affiliations:** 1 Institute of Anatomy, Division of Cell Biology, University of Zurich, Winterthurerstrasse 190, 8057, Zurich, Switzerland; 2 Institute of Physiology, Center for Integrative Human Physiology, University of Zurich, Winterthurerstrasse 190, 8057, Zurich, Switzerland

## Abstract

**Background:**

Cytotoxic T lymphocytes (CTL) and natural killer (NK) cells play an essential role in the host defence against intracellular pathogens such as *Listeria*, and *Mycobacteria*. The key mediator of bacteria-directed cytotoxicity is granulysin, a 9 kDa protein stored in cytolytic granules together with perforin and granzymes. Granulysin binds to cell membranes and is subsequently taken up via a lipid raft-associated mechanism. In dendritic cells (DC) granulysin is further transferred via early endosomes to *L. innocua*-containing phagosomes were bacteriolysis is induced. In the present study we analysed the role of perforin in granulysin-induced intracellular bacteriolysis in DC.

**Results:**

We found granulysin-induced lysis of intracellular *Listeria *significantly increased when perforin was simultaneously present. In pulse-chase experiments enhanced bacteriolysis was observed when perforin was added up to 25 minutes after loading the cells with granulysin demonstrating no ultimate need for simultaneous uptake of granulysin and perforin. The perforin concentration sufficient to enhance granulysin-induced intracellular bacteriolysis did not cause permanent membrane pores in *Listeria*-challenged DC as shown by dye exclusion test and LDH release. This was in contrast to non challenged DC that were more susceptible to perforin lysis. For Listeria-challenged DC, there was clear evidence for an Ca^2+ ^influx in response to sublytic perforin demonstrating a short-lived change in the plasma membrane permeability. Perforin treatment did not affect granulysin binding, initial uptake or intracellular trafficking to early endosomes. However, enhanced colocalization of granulysin with listerial DNA in presence of perforin was found by confocal laser scanning microscopy.

**Conclusion:**

The results provide evidence that perforin increases granulysin-mediated killing of intracellular *Listeria *by enhanced phagosome-endosome fusion triggered by a transient Ca^2+ ^flux.

## Background

CTL and NK cells play an essential role in the host defence against intracellular microbial pathogens like *Listeria monocytogenes *[1] or *Mycobacteria tuberculosis *[2]. Several mechanisms are involved in clearance of intracellular bacteria including cytokine release [3], apoptosis induction of the host cell [4], and directly executed antimicrobial activity of CTL. The direct antibacterial activity of CTL is mediated by granulysin [5], a 9 kDa protein discovered by subtractive hybridization of late activated T-cells [6,7]. Recombinant granulysin proved to exhibit a broad spectrum of antimicrobial activity against bacteria, fungi and parasites [8]. We recently demonstrated that granulysin enters human DC in a lipid raft-associated mechanism and gains access to *L. innocua *located in phagosomes to induce bacteriolysis [9]. However, intracellular bacteria were never fully eradicated by treatment of the DC with granulysin only. As granulysin is stored in granules of CTL together with other lytic proteins such as perforin, the interaction of both proteins in killing intracellular bacteria is of great interest. Perforin shares homology with the terminal complement components and can thus multimerize in eukaryotic membranes to form pores of a diameter of 50 nm or less. These perforin pores were initially thought to allow passage of lytic granule components, especially granzymes, to the target cell cytosol [10-13]. The discovery of granzyme B to be endocytosed independently of perforin as well as the induction of target cell apoptosis by granzyme B and perforin without detectable leakage of the plasma membrane questioned the pore formation model [14,15]. In contrary, Keefe et al. demonstrated a transient Ca^2+ ^flux in HeLa cells in response to sublytic perforin concentrations indicating short-lived membrane pores that are immediately repaired by the cellular wound healing system [16].

The role of perforin in granulysin-mediated intracellular bacteriolysis is contradictory. Stenger et al. demonstrated that perforin was necessary for granulysin-mediated killing of intracellular *M. tuberculosis *in macrophages [8]. On the other hand, cell based *in vitro*-studies showed that killing of intracellular *Mycobacteria *by CD4^+ ^and CD8^+ ^cytotoxic T cells occurred independently of perforin [17]. The role of perforin in delivering granulysin was further questioned in a study using *M. leprae *also residing within phagosomes of host cells. Ochoa et al. demonstrated by phenotyping cells in dermal granuloma of leprosy lesions that the majority of cells containing granulysin were CD4^+ ^and CD3^+^, but negative for perforin [18].

Using our proven model system, we investigated the interaction of perforin and granulysin in human monocyte-derived DC as hosts harbouring *L. innocua*, a gram positive, apathogenic bacterium ubiquitously distributed in our environment [19]. Influence of perforin on granulysin binding, uptake and trafficking was visualized and quantified as well as correlated to the lysis of intracellular *L. innocua*. It was further examined if stable plasma membrane pore formation and/or short lived changes in the plasma membrane integrity allowing transient Ca^2+ ^fluxes is a prerequisite for the mediation of the perforin effect.

## Results

### Perforin enhances significantly granulysin-mediated lysis of *L. innocua *in DC

**Figure 1:**
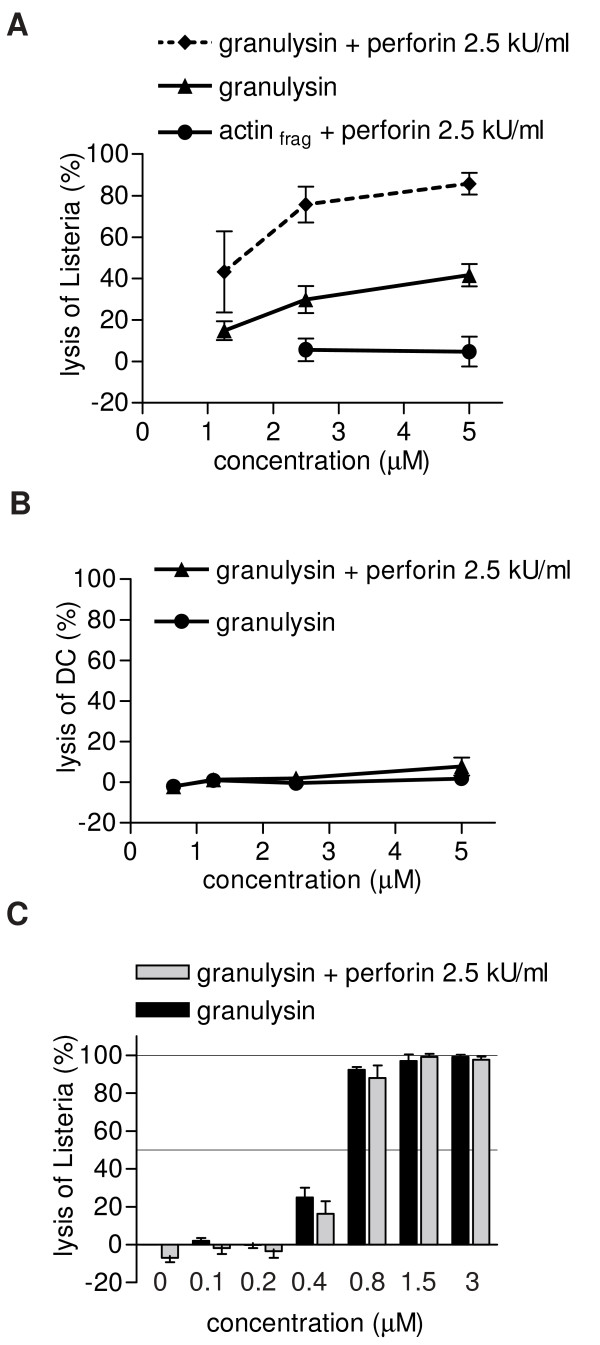
**Effect of granulysin and perforin treatment on *L. innocua *in human DC, DC viability, and *L. innocua *in suspension**. (A) *L. innocua*-challenged DC were incubated for 3 hours with granulysin or actin_frag _as a control in varying concentrations in presence or absence of 2.5 kU/ml perforin at 37°C. After granulysin incubation, the cells were lysed in ice-cold water and *Listeria *viability was assessed in CFU assays. Mean values and SD of four independent experiments are presented. (B) *L. innocua*-challenged DC were incubated with granulysin at indicated concentrations in presence or absence of 2.5 kU/ml perforin for 3 hours at 37°C. After the incubation LDH content was measured in the supernatant and specific lysis was calculated. Mean values and SD of three independent experiments are presented. (C) *L. innocua *was incubated with granulysin in indicated concentrations +/- 2.5 kU/ml perforin for 3 hours at 37°C. After the granulysin incubation *Listeria *viability was calculated from bacterial growth curves obtained by turbidimetry.

In a previous study we showed that about 40% of *L. innocua *located intracellular in human DC were killed by granulysin [9]. As granulysin is stored in cytolytic granules together with perforin, we evaluated the influence of simultaneously applied perforin in granulysin-induced bacteriolysis in human DC. Treatment of *L. innocua*-challenged DC with various concentrations of granulysin and a constant perforin-concentration of 2.5 kU/ml for 3 hours at 37°C reduced the viability of the intracellular bacteria dose-dependently as tested in colony forming unit (CFU) assays (Fig. 1a). At a granulysin-concentration of 2.5 μM together with 2.5 kU/ml perforin more than 75% of the intracellular *Listeria *were lysed. Furthermore, perforin significantly enhanced intracellular bacteriolysis at granulysin concentrations of 1.25 (p = 0.05), 2.5 and 5 μM (for both p < 0.01, Fig. 1A). When infected DC were treated with granulysin +/- perforin at 4°C or with actin_frag _+/- perforin no killing of intracellular bacteria was observed (data not shown and [9]).

In order to exclude bacterial loss due to detachment of perishing host cells, the viability of *Listeria*-challenged DC during incubation with granulysin alone or in combination with perforin was assessed in LDH release assays. Granulysin in all tested concentrations did not cause a significant LDH release from DC (Fig. 1b). Incubation of granulysin (2.5 μM) with perforin (2.5 kU/ml) resulted in DC lysis of less than 2% (Fig. 1b). Therefore, 2.5 μM granulysin and 2.5 kU/ml perforin were considered to be sublytic to host cells and were used in all subsequent experiments. Perforin incubated alone in concentrations up to 10 kU/ml was found to have no lytic activity on *Listeria*-challenged DC (LDH release < 7%). This result further indicates that perforin in the concentrations applied does not lead to an increase in membrane permeability sufficient for a detectable release of LDH from treated DC.

*L. innocua *in suspension treated with granulysin showed dose-dependently a decrease in viability as calculated from bacterial growth curves. At granulysin concentrations down to 1.5 μM more than 90% of the bacteria were killed (Fig. 1c). The median effective dose was determined in the concentration range of 0.4 to 0.8 μM. Below 0.2 μM killing activity decreased to background level. Unlike to the killing efficiency of intracellular *L. innocua*, simultaneous perforin application at a concentration of 2.5 kU/ml did not further decrease bacteriaviability in suspension. Furthermore, perforin applied alone in concentrations up to 10 kU/ml did not affect *Listeria *viability (data not shown).

Taken together, granulysin-mediated lysis of intracellular *L. innocua *in human DC is significantly enhanced by perforin.

### Granulysin and perforin can be sequentially added to induce enhanced intracellular bacteriolysis

**Figure 2:**
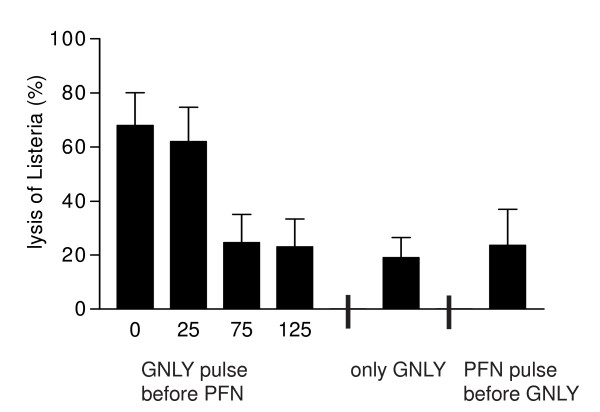
**Granulysin and perforin can be sequentially added to induce enhanced intracellular listeriolysis**. *L. innocua*-challenged DC were pulsed for 15 minutes with 2.5 μM granulysin and after medium replacement were then treated at indicated time points with perforin at a concentration of 2.5 kU/ml at 37°C for an overall experimental duration of 180 minutes. Alternatively, *L. innocua*-challenged DC were pretreated for 15 minutes with perforin and after medium replacement were then incubated with granulysin at a concentration of 2.5 μM at 37°C for 180 minutes. After the incubation, the cells were lysed in ice-cold water and *Listeria *viability was calculated from bacterial growth curves. Mean values and SD of four independent experiments are presented.

Using pulse-chase experiments, we evaluated whether granulysin and perforin must be simultaneously present or can be sequentially added for enhanced intracellular bacteriolysis. For this purpose DC were first pulsed with 2.5 μM granulysin for 15 minutes at 4°C and, after medium replacement, further incubated at 37°C to allow endocytosis of granulysin. At indicated time points during the chase period, DC were treated with perforin at a concentration of 2.5 kU/ml at 37°C for an overall experimental duration of 180 minutes. When perforin treatment followed immediately or up to 25 minutes after the granulysin pulse, a significant increase of bacteriolysis was detected as compared to samples incubated without perforin (Fig. 2). Perforin application 75 minutes after pulsing the DC with granulysin did not lead to a significant enhancement of intracellular bacteriolysis, even with prolonged experimental duration to obtain a perforin incubation time 180 minutes (data not shown). Likewise, first pretreating with perforin for 15 minutes at 37°C before medium replacement and subsequently incubating with granulysin for 180 minutes did not significantly enhance intracellular bacteriolysis (Fig. 2).

### Sublytic perforin does not result in stable plasma membrane pores

**Figure 3:**
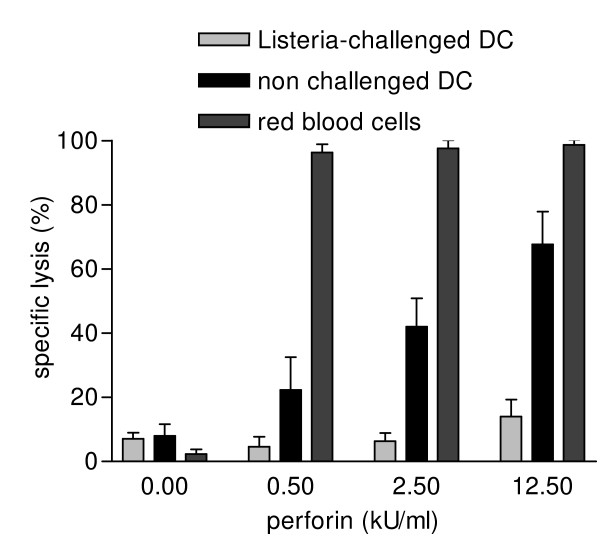
**Sublytic perforin does not result in stable pores but induces a transient Ca^2+ ^flux**. *L. innocua*-challenged DC or non challenged DC were incubated with various concentrations of perforin for 45 minutes at 37°C in the presence of 2 μM EthD-2 in RPMI. After fixation, the percentage of cells that had incorporated the dye was determined by counting three independent experiments by confocal microscopy. As a quality control in parallel experiments, an equal amount of red blood cells were perforin treated for 45 minutes at 37°C before assessing hemolysis.

Perforin was described to induce pore formation in eukaryotic cell membranes [12,20]. Therefore, we investigated the pore forming capability of perforin on *L. innocua*-challenged human DC using LDH release assays (Fig. 1b). Since LDH is a rather large molecule (140 kDa) that might not diffuse by passive efflux through transient perforin pores we chose a detection system using the cell-impermeant nucleic acid stain ethidium homodimer-2 (EthD-2; 1.2 kDa). Perforin in a concentration of 2.5 kU/ml used for granulysin-mediated bacteriolysis caused no raise in membrane permeability for EthD-2 in *Listeria*-challenged DC (Fig. 3). Just a moderate increase in EthD-2 positive DC up to 14% was detected at a perforin concentration of 12.5 kU/ml. As *Listeria*-challenged DC demonstrated to be rather refractory to perforin lysis, we also tested the susceptibility of unchallenged DC to perforin lysis. We found significant lysis of unchallenged DC already at a perforin concentration of 0.5 kU/ml (23.7%, p = 0.035) which dose dependently increased up to 67.7% at a concentration of 12.5 kU/ml. To demonstrate the quality of the perforin preparation, the hemolytic activity was always tested in parallel experiments (Fig. 3). These results clearly indicate that *Listeria *challenge of DC alters their susceptibility to perforin lysis.

Stable perforin membrane pores could be excluded to be a major factor for the enhancement effect in granulysin-mediated bacteriolysis.

### Sublytic perforin induces a transient Ca^2+ ^influx

**Figure 4:**
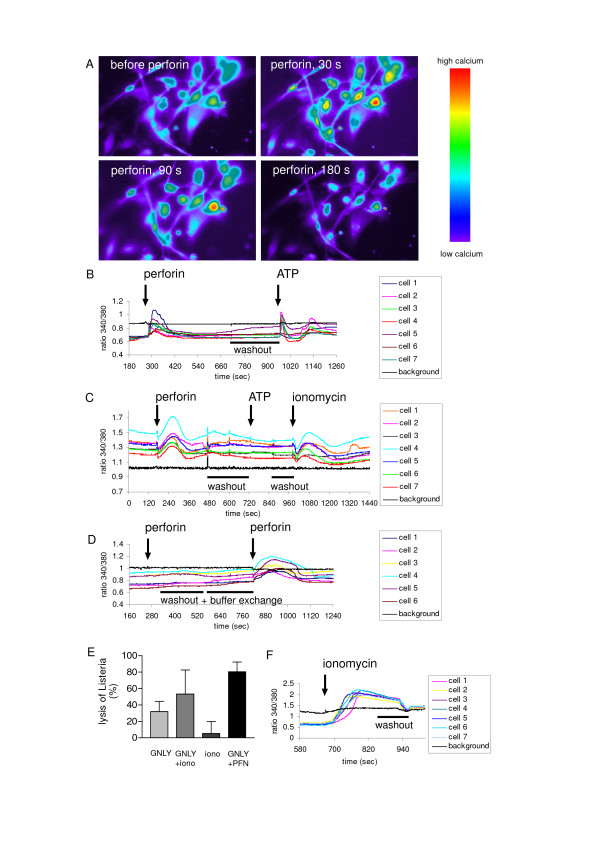
**Sublytic perforin induces a transient Ca^2+ ^influx**. (A) – (D), and (F), DC were loaded with Fura-2 for 15 minutes prior to the incubation with perforin in a concentration of 2.5 kU/ml. At indicated time points fluorescent images (Excitation 340 nm; Emission 525 nm) were recorded (A). The excitation ratio 340/380 nm was monitored over the whole experimental period (B). Some cultures were pretreated with TMB-8 (10 μM) and dantrolene (25 μM) to block intracellular Ca^2+ ^mobilization (C). (D), DC were equilibrated in a low Ca^2+ ^(1.3 μM) buffer before a first exposure to perforin, after washout and buffer exchange to normal Ca^2+ ^conditions (1.3 mM) the cells were again treated with perforin. ATP and ionomycin were used as positive controls for Ca^2+ ^mobilization from intracellular or extracellular sources. One representative measurement of more than six independent experiments is presented. (E), *L. innocua*-challenged DC were incubated for 3 hours with granulysin in presence or absence of 2.5 kU/ml perforin or 1 μM ionomycin at 37°C or were incubated with ionomycin alone. After the incubation, the cells were lysed in ice-cold water and *Listeria *viability was calculated from bacterial growth curves. Mean values and SD of three independent experiments are presented. (F), to demonstrated typical Ca^2+ ^responses to ionomycin, DC were pretreated with Fura-2 for 15 minutes prior to the incubation with ionomycin (1 μM). One representative measurement out of four independent experiments is presented.

The existence of short-lived changes of the plasma membrane integrity still could not be excluded, since passive diffusion of EthD-2 might not be fast enough for intracellular accumulation of detectable levels of the dye. As perforin was shown to trigger a transient Ca^2+ ^flux in HeLa cells [16], intracellular Ca^2+ ^in response to perforin and/or granulysin was assessed in DC. For this purpose *Listeria*-challenged DC were loaded with the Ca^2+ ^sensitive dye Fura-2 before treatment with perforin and/or granulysin. The application of 2.5 kU/ml perforin on DC resulted in an immediate and transient rise of the intracellular Ca^2+ ^concentration which recovered completely within the next 3 minutes (Fig. 4a and 4b). The intensity of the response varied among the cells but an increase of the intracellular Ca^2+ ^concentration was measured in every single cell at the indicated perforin concentration. The transient Ca^2+ ^flux did not disturb critically the viability or even the Ca^2+ ^homeostasis in the cells as consecutive treatment with ATP (1 mM), which triggers Ca^2+ ^release from intracellular sources [21], resulted in the typical biphasic curve of the excitation ratio. Granulysin incubated alone at concentrations up to 5 μM did not change the intracellular Ca^2+ ^level and also simultaneous granulysin and perforin treatment did not increase the response compared to perforin treatment alone (data not shown).

To investigate the source of the Ca^2+ ^responsible for the transient flux, *Listeria*-challenged DC were pretreated with the established intracellular calcium channel blockers TMB-8 (10 μM) and dantrolene (25 μM) [22-25] prior to the perforin incubation. Perforin in a concentration of 2.5 kU/ml induced a transient Ca^2+ ^flux in the pretreated DC (Fig. 4c). Aiming to verify the inhibition of the Ca^2+ ^mobilization from intracellular sources, the DC were subsequently treated with ATP. In TMB-8/dantrolene-pretreated DC ATP did not cause a Ca^2+ ^response, in contrast to the Ca^2+ ^ionophor ionomycin (1 μM), which resulted in a Ca^2+ ^rise (Fig. 4c). The effect of perforin in low Ca^2+ ^buffer was also tested (Fig. 4d). For this purpose *Listeria*-challenged DC were equilibrated in a low Ca^2+ ^(1.3 μM) buffer before exposure to perforin, which did not result in a Ca^2+ ^response. Only after washout and buffer exchange to normal Ca^2+ ^conditions (1.3 mM), a Ca^2+ ^flux could be provoked by perforin. Comparable to the EthD-2 results above, non *Listeria*-challenged DC were by a factor 5–10 more sensitive to perforin triggered Ca^2+ ^fluxes (data not shown).

The ability of granulysin to induce Ca^2+ ^fluxes was investigated by incubating granulysin alone at concentrations up to 5 μM, which did not change the intracellular Ca^2+ ^level. Furthermore, simultaneous granulysin and perforin treatment did not increase the response compared to perforin treatment alone (data not shown). These result provide clear evidence that sublytic perforin triggers a transient Ca^2+ ^influx in *Listeria*-challenged DC. Finally, the role of the transient Ca^2+ ^rise in the granulysin-mediated intracellular bacteriolysis was investigated by the comparison of the enhancing effect of perforin and ionomycin. Ionomycin demonstrated a clear tendency to enhance granulysin-mediated bacteriolysis in all tested samples but due to broader distribution of the single values an overall significance was barely not achieved (p = 0.063) in contrast to the effect of perforin (p < 0.001) (Fig. 4e). The Ca^2+ ^rise induced by ionomycin was furthermore distinguishable from that triggered by perforin as the response was prolonged and the recovery incomplete (Fig. 4f).

### Perforin does not influence granulysin binding and initial uptake in DC

**Figure 5:**
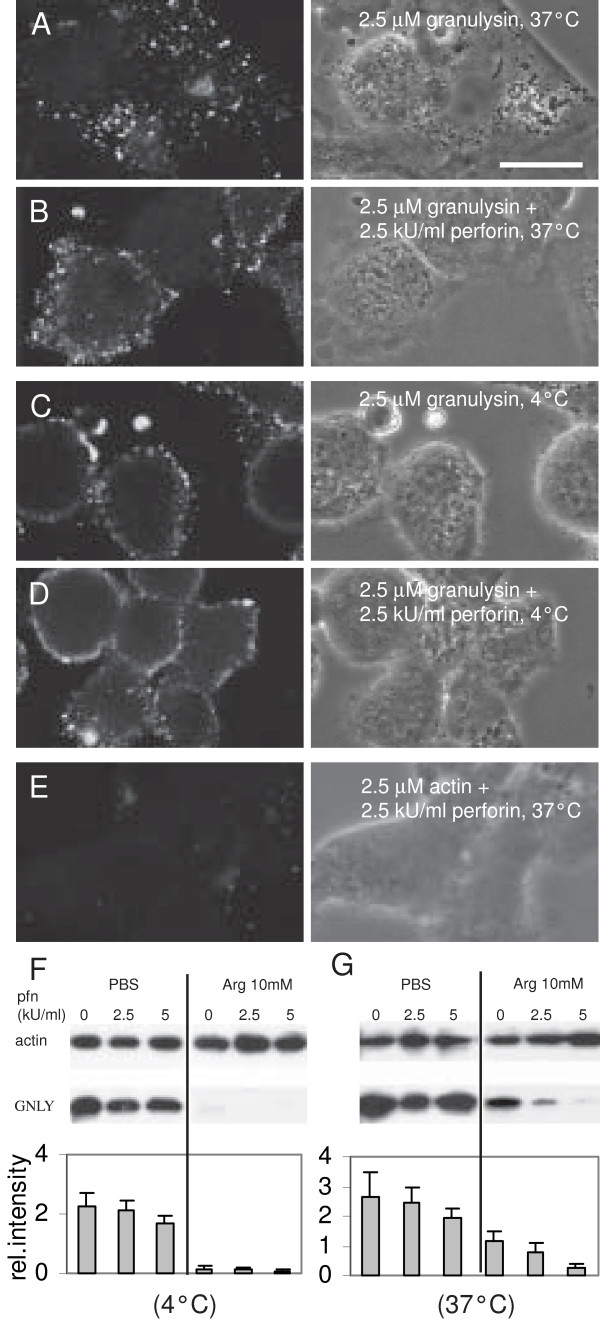
**Influence of perforin on binding and uptake of granulysin in DC**. *L. innocua*-challenged DC were incubated for 45 minutes with 2.5 μM granulysin (A), or with 2.5 μM granulysin and 2.5 kU/ml perforin (B) at 37°C, or at 4°C (C and D). As a control, cells were treated with 2.5 μM actin_frag _and 2.5 kU/ml perforin (E). After the incubation, the cells were fixed and stained with the anti-His Ab for CLSM. Representative phase contrast (right panels) and immunofluorescence images (left panels) are shown, bar = 8 μm. *L. innocua*-challenged DC were treated for 45 minutes with 2.5 μM granulysin and indicated concentrations of perforin at 4°C (E) or 37°C (F). After the incubation the DC were washed with PBS or with 10 mM arginine in PBS before lysis with 0.5% Triton-X-100 in PBS. The level of bound or internalized granulysin was analysed by Western blotting. Granulysin was detected with the anti-His mAb. As a reference, cellular actin was detected using an anti-β-actin mAb. Relative intensity of the bands was determined using Image J software. Mean values and SD of four independent experiments are presented.

To elucidate the underlying mechanism of the enhancing effect of perforin, the impact of perforin on granulysin binding and initial uptake was studied. By confocal laser scanning microscopy a spot like pattern of granulysin was detected in *L. innocua*-challenged DC which was clearly localized within the cells when incubated at 37°C (Fig. 5a). After incubation at 4°C, granulysin labeling was found attached to the cell membrane and no significant uptake occurred (Fig. 5c). A similar staining pattern of binding and initial uptake was detected after granulysin treatment of non-infected DC (data not shown and [9]). No difference in the localization of granulysin or intensity of the staining could be found in cells that were treated with granulysin and perforin (Fig. 5b and 5d) compared to cells that were exposed only to granulysin. When the DC were treated with the His-tagged control protein actin_frag _in presence of perforin at 37°C, no bound or internalized protein could be detected demonstrating both the membrane binding properties of granulysin as well as the specificity of the anti-His Ab (Fig. 5e).

To quantify the influence of perforin on granulysin binding and uptake western blot analysis was performed. Following an adapted protocol of Shi et al. [26], externally bound granulysin was removed by washing with 10 mM arginine to quantify the internalized granulysin fraction. After incubation of DC with granulysin at a concentration of 2.5 μM at 4°C, the arginine washing step removed over 95% of the bound granulysin compared to PBS washing (Fig. 5f). After incubation at 37°C 44% of the total granulysin signal remained detectable following the arginine incubation and was therefore considered as the internalized fraction (Fig. 5g). Co-incubation of perforin in concentrations of 2.5 and 5 kU/ml did not significantly affect the binding of granulysin to DC membranes (Fig. 5f). Granulysin uptake tended to decrease in response to 2.5 kU/ml perforin but the decline was not statistically significant regarding all independent experiments (p = 0.16). A perforin concentration of 5 kU/ml significantly reduced granulysin uptake in human DC (p = 0.009, Fig. 5g).

### Perforin does not affect granulysin transfer to early sorting endosomes

**Figure 6:**
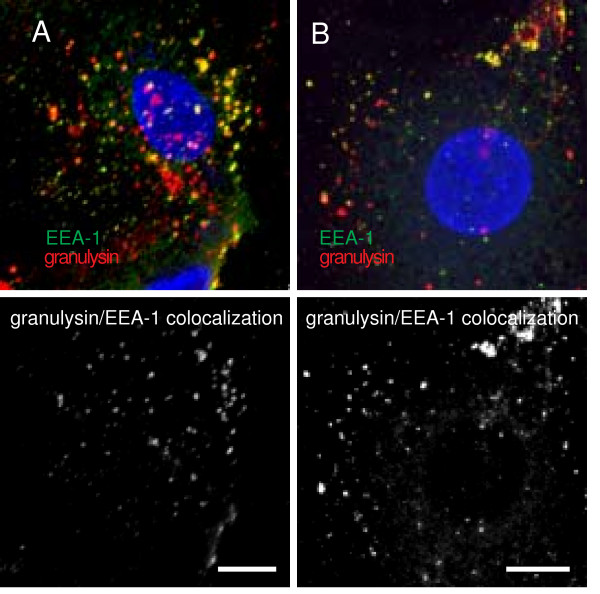
**Effect of perforin on granulysin transfer to early endosomes in DC**. *L. innocua*-challenged DC were incubated with 2.5 μM granulysin (A) or with 2.5 μM granulysin and 2.5 kU/ml perforin (B) for 45 minutes at 37°C. After fixation, the cells were stained with an anti-His Ab and with a polyclonal Ab recognizing the early endosomal antigen 1 (EEA-1). Nuclear and bacterial DNA was labeled with DAPI (blue), early endosomes are marked in green, and granulysin is depicted in red. Colocalization of the EEA-1 (green) and granulysin (red) was additionally calculated and visualized using Imaris software (lower row), bar = 8 μm.

Following uptake granulysin is found in early sorting endosomes [9]. To evaluate whether perforin affects this step in granulysin trafficking double labeling of a molecule specific for the endocytic compartment (early endosomal antigen-1; EEA-1) and granulysin was performed. Granulysin was found colocalized with EEA-1 after incubating *L. innocua *infected DC with granulysin for 45 minutes at 37°C in steady-state (Fig. 6a). Simultaneous perforin treatment (2.5 kU/ml) did not affect the intensity of granulysin labeling in the EEA-1 positive organelles (Fig. 6b). Using Imaris software colocalization images of EEA-1 and granulysin were calculated (Fig. 6a and 6b, lower row). Quantification of these images concerning the granulysin fraction found in the EEA-1 positive vesicles revealed no significant difference between the DC that were only granulysin treated or those that were granulysin and perforin treated (data not shown). Taken together, these results indicate that perforin does not influence the amount of granulysin residing in early sorting endosomes of DC.

### Perforin enhances granulysin transfer to *Listeria*-containing phagosomes

**Figure 7:**
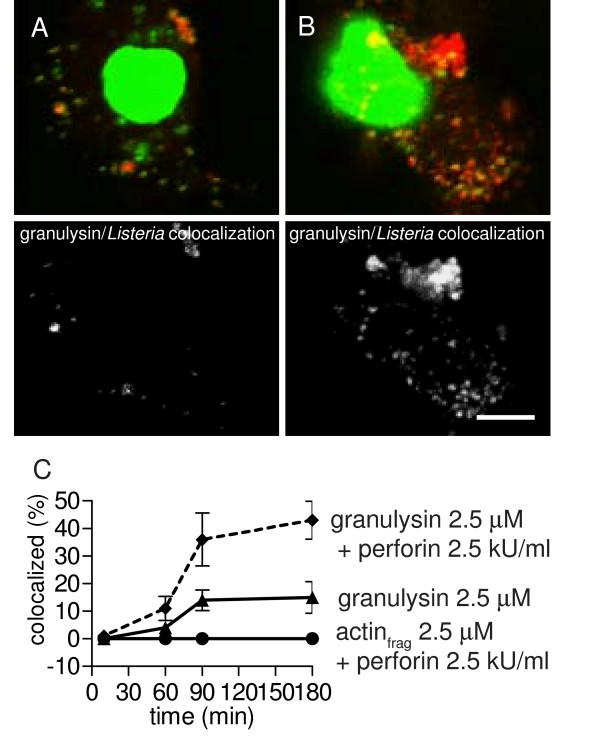
**Effect of perforin on granulysin transfer to *L. innocua*-containing phagosomes**. Representative images of *L. innocua*-challenged DC that were incubated with 2.5 μM granulysin (A) or with 2.5 μM granulysin and 2.5 kU/ml perforin (B) for 3 hours at 37°C. After fixation, the cells were stained with an anti-His Ab. Nuclear and bacterial DNA was labeled with DAPI (green), and granulysin is depicted in red, colocalization of the DAPI and granulysin was calculated and visualized using Imaris software (lower row), bar = 8 μm. At indicated time points colocalization of bacterial DNA with the granulysin staining was quantified in four independent experiments (C) Mean values and SD of colocalization in all evaluated cells per time point of four independent experiments are presented.

Granulysin has to gain access to *L. innocua *located in phagosomes of the DC to successfully mediate intracellular bacteriolysis. In order to study granulysin transfer to phagosomes, *L. innocua*-challenged DC were treated for the indicated time periods with 2.5 μM granulysin in presence or absence of 2.5 kU/ml perforin and stained with the anti-His mAb. In high resolution images the degree of colocalization of DAPI labeled bacterial DNA with granulysin at indicated time points was determined. Representative images of granulysin and granulysin/perforin treated DC are shown in Fig. 7a and 7b, respectively, merged images in the upper row, colocalization micrographs in the lower row. Colocalization of bacterial DNA and granulysin was already observed to some extent when DC were only granulysin treated but more colocalization was found in DC that were simultaneously granulysin and perforin treated (Fig. 7a). Quantification of these images revealed that after 90 minutes a plateau was reached with 14% of the *Listeria *colocalized with the granulysin staining (Fig. 7c). When DC were incubated with granulysin and perforin we found after 90 minutes 36% of the listerial DNA colocalized with granulysin that even slightly increased to 43% after 180 minutes. Therefore, the overall transfer of granulysin to *Listeria *containing phagosomes is increased in response to perforin.

## Discussion

Recently we have shown a perforin-independent pathway of granulysin-mediated bacteriolysis in human DC [9]. Granulysin proved to bind and to be endocytosed in a lipid raft-associated mechanism followed by transfer to *L. innocua*-containing phagosomes where bacteriolysis was induced. The killing of intracellular *Listeria *by granulysin was shown to be dose-dependent, but full eradication of the bacteria was not achieved at any applied concentration.

In the current study we were able to demonstrate a significant and robust enhancing role of perforin in granulysin-mediated lysis of intracellular *L. innocua *in human DC. This enhancing effect could neither be explained by a simple cell loss due to perforin toxicity against the host cells nor by a direct antimicrobial activity of perforin. The observed role of perforin in our experimental system is in agreement with data of other groups describing perforin as a delivering or triggering molecule [8,13,14,27]. For the particular interaction of recombinant granulysin and purified perforin there is just a single study up to now that showed perforin to be essential for granulysin-mediated lysis of *M. tuberculosis *in human macrophages [8]. However, these results are not fully consistent with the results we obtained in our experimental system using a non pathogenic strain of *L. innocua *that is killed to some extent by granulysin alone. Furthermore, *L. innocua *is transferred to lysosomes and killed within 48 hours by the DC itself (data not shown). Mycobacteria like *L. innocua *used in our system reside in phagosomes of the host cell [28] but in contrast to *L. innocua *virulent *Mycobacteria *have evolved mechanisms to prevent phagosome maturation, especially the fusion with lysosomes to form the lethal phagolysosomes [29]. This active interference of virulent *Mycobacteria *with the endocytic machinery of the host cell might provide an explanation that granulysin alone does not gain access to the specialized *Mycobacteria*-containing phagosomes and therefore is not capable of killing the bacteria without perforin.

The triggering role of perforin in the granzyme B-induced apoptosis is, in contrast to the mechanism of granulysin induced bacteriolysis, extensively investigated [14,27,30], but underlying mechanisms are still under debate [15,26,31]. Similar to granulysin, granzyme B is endocytosed independently of perforin but for induction of the target cell apoptosis it has to gain access to the cytosol. For this delivery step co-treatment of the target cell with perforin is essential [14]. The interaction of granzyme B and perforin is not fully comparable to the findings in our experimental system as we did not find any indication for cytosolic delivery of granulysin in response to perforin but observed enhanced vesicular targeting to *L. innocua *containing phagosomes.

As perforin was originally described as a pore forming protein we tested its pore forming capability in DC using LDH release assay as well as by the ability to promote the entrance of membrane impermeant EthD-2 or of Ca^2+^. Perforin in concentrations sufficient for enhanced lysis of intracellular *L. innocua *resulted in a transient Ca^2+ ^increase but not in a detectable release of LDH or accumulation of EthD-2 in *Listeria*-challenged DC. The source of the Ca^2+ ^was demonstrated to be the extracellular space as blocking intracellular Ca^2+ ^channels had not effect on Ca^2+ ^waves triggered by perforin or ionomycin. This is further supported by low Ca^2+ ^buffer conditions which abolished perforin induced Ca^2+ ^fluxes. As perforin activity is dependent on free Ca^2+^, this result might also refer to disturbed perforin membrane binding and multimerization. In our experimental system, no evidence could be provided for a granulysin induced Ca^2+ ^release from intracellular stores as this was reported for higher concentration of granulysin in tumor cells (50 μM, [32]). These results clearly indicate that sublytic perforin triggers a short-lived disturbance of the plasma membrane permeability not sufficient for significant entrance of a small fluorescent dye but sufficient for a transient Ca^2+ ^influx.

It was coincidentally shown that unchallenged DC were more susceptible to perforin lysis than *Listeria*-challenged DC. This interesting phenomenon might be explained by recent data showing elevated cathepsin B expression in Toll-like receptor activated DC [33]. Cathepsin B was on the other hand suggested to protect CTL and NK cells from autolysis after degranulation [34]. The protection effect of cathepsin B was called recently into question [35] and as no further experimental evidence is provided, about mechanisms leading to higher resistance of *Listeria*-challenged DC against perforin lysis can only be speculated.

In pulse-chase experiments it was demonstrated that enhanced bacteriolysis was still observed when DC were perforin treated up to 25 minutes subsequent to incubation with granulysin. On the other hand, pretreatment of DC with perforin prior to granulysin-mediated listeriolysis had no enhancing effect. These results suggest that stable perforin pores seem not to play a role in promoting augmented passive diffusion of granulysin into target cells. Therefore, the classical pore formation model of perforin allowing delivery of lytic proteins by an increase of membrane permeability is not a major mechanism in granulysin uptake and the induction of enhanced intracellular bacteriolysis. There is a remarkable drop in the enhancing effect, if perforin is first added 75 minutes after pulsing the cells with granulysin. From this result coendocytosis of granulysin and perforin, at least to some extent, can not be excluded to play a role in the enhanced killing of *L. innocua *in DC. Coendocytosis of granzyme B and perforin to a certain amount was shown to be critical for the induction of target cell apoptosis [26]. The biological role of the transient Ca^2+ ^flux remains to be clarified. Recently, it was shown in HeLa cells that a sublytic perforin concentration triggers via Ca^2+ ^signaling a rapid membrane repair response in the sense of a cellular wound healing system [16]. This process includes the rapid removal of the damaged membrane area from the surface as well as the resealing with intracellular vesicles [36-39]. As a result of this increased membrane turnover in response to perforin, granzyme B is accelerated and augmented endocytosed by target cells [16]. Part of this response is exuberant homotypic and heterotypic membrane fusion, which accelerates phagosome maturation [36]. In another study it was furthermore demonstrated that the inhibition of Ca^2+ ^signaling by *Mycobacterium tuberculosis *is associated with reduced phagosome-lysosome fusion and crucial for the prolonged survival of the pathogen within phagosomes of macrophages [40].

To further reveal the mechanism of perforin as an enhancing molecule we studied the impact of perforin on all critical steps of granulysin trafficking in DC, i.e. the binding and initial uptake, the transfer to early sorting endosomes and finally the transmission to *L. innocua*-containing phagosomes. Sublytic concentrations (2.5 kU/ml) of perforin had no apparent effect on granulysin binding, uptake or transfer to early sorting endosomes. These results are not consistent with a recent study showing that sublytic concentrations of perforin triggers accelerated and enhanced endocytosis of granzyme B as well as transfer to large EEA-1-positive vesicles in HeLa cells [16]. In our experimental system granulysin binding or initial uptake is not the limiting factor for the enhanced induction of bacteriolysis in DC in response to perforin. To the contrary, perforin at a concentration of 5 kU/ml significantly inhibited granulysin uptake in DC. As this perforin concentration is sublytic to DC, the finding of inhibited uptake was unexpected. Because no experimental data is available, it can only be speculated if sublethal damage to DC by perforin inhibits complex cellular processes like endocytosis.

In contrast to binding and initial uptake vesicular targeting of granulysin to *L. innocua*-containing phagosomes was significantly enhanced by perforin treatment as an indication for improved endosome-phagosome fusion. Early endosomes like all other endocytic organelles can fuse with phagosomes and this fusion process is Ca^2+ ^sensitive [41-44]. We found furthermore some evidence that elevated intracellular Ca^2+ ^indeed plays a role as a signal event promoting enhanced granulysin induced bacteriolysis. The Ca^2+ ^ionophor ionomycin showed a tendency to increase the granulysin mediated intracellular listeriolysis. The mechanism of the intracellular Ca^2+ ^elevation induced by ionomycin is presumably quite different from that induced by perforin which is also reflected in a different shape of the Ca^2+ ^waves with prolonged and incomplete recovery phases. This might explain the inefficient enhancement of ionomycin in comparison to perforin. On the other hand, it is not excluded that perforin triggers additional signalling cascades apart from Ca^2+ ^that are not activated by ionomycin. Finally, we may speculate that sublytic perforin triggers the membrane repair response via Ca^2+ ^signalling. In the course of the membrane repair response endosome-phagosome fusion and thus transmission of granulysin to phagosomes is more likely resulting in enhanced bacteriolysis.

## Conclusion

In conclusion, perforin promotes enhanced bacteriolysis by granulysin not by the formation of stable pores that allow passive diffusion of granulysin but rather by an increase in endosome-phagosomes fusion triggered by an intracellular Ca^2+ ^rise.

## Methods

### Production of recombinant granulysin

Recombinant granulysin was produced as previously described [9]. Briefly, a construct corresponding to NKG5 from G 63 to D 132 was cloned in pET28a (Novagen, Inc., WI, USA) containing a c-terminal hexahistidine fusion tag as well as a factor Xa protease cleavage site. A fragment of human β-actin was used as a control protein. Proteins were expressed in Escherichia coli BL21 (DE3) additionally transformed with the chloramphenicol-resistant plasmid, pRARE (Novagen). After lysis of bacteria, granulysin was purified via nickel affinity chromatography, further renatured according to the protocol of Ernst et al. (18) and finally purified using Sep-Pak Vac 6 cc (1 g) C18 cartridges (Waters, Milford, MA, USA).

### Purification of perforin and determination of hemolytic activity

Perforin was purified from YT Indy cells according to the protocol of Froelich et al. [45]. Briefly, YT cells were collected by centrifugation, washed twice in Hank's buffered salt solution (HBSS), and resuspended at 10^8 ^cells/ml in ice cold relaxation buffer (10 mM PIPES, 0.1 M KCl, 3.5 mM MgCl_2_, 1 mM ATP, 1.25 mM EGTA, 0.05% BSA, pH 6.8). The cells were disrupted in a nitrogen cavitation bomb (YEDA Scientific Instruments, Rehovot, Israel) at 35 bar at 4°C for 10 minutes. The resulting lysate was centrifuged at 400 × g for 7 minutes and the postnuclear supernatant at 15000 × g for 15 minutes to yield the granule pellet. The granule pellet was extracted by mixing with 1 M NaCl in 20 mM Na-acetate, pH 4.5 containing 2 mM EDTA for 60 minutes at 4°C. After a freeze-thaw cycle the extract was centrifuged at 8,500 × g for 10 minutes. The supernatant was applied to a Econo-Pac 10 DG column (BioRad, Hercules, CA, USA) and eluted with 1 M NaCl, 20 mM HEPES, 10% betaine. The granule extract was purified using cobalt affinity chromatography and eluted by a linear imidazole gradient (0–0.2 M). All gradient fractions were tested for hemolytic activity using a protocol adapted from Henkart et al. [46]. Briefly, dilutions of the fractions in assay buffer (10 mM Hepes, 0.15 M NaCl, and 0.1% BSA, pH 7.5) were incubated with an equal volume of 0.2% human red blood cells (in assay buffer containing 5 mM CaCl_2_) at 37°C for 20 min in round bottom microtiter plates (Nunc, Rochester, NY, USA). The microtiter plates were centrifuged at 500 × g for 6 min and the supernatants transferred to a second flat bottom microtiter plate. The hemoglobin released into the supernatant was detected with the microplate reader (Spectra MAX 340, Molecular Devices, Sunnyvale, CA, USA) at a wavelength of 420 nm. The fractions containing the highest hemolytic activity were combined and concentrated by ultrafiltration (Centricon, 30 kDa MWCO, Millipore, Billerica, MA, USA) in the presence of EDTA and BSA to yield final concentrations of 2 mM, and 100 mg/ml, respectively. 1 hemolytic unit (U) was defined as the amount resulting in 50% lysis of 0. 1% red blood cells in a volume of 0.2 ml, corresponding to a total amount of 2000000 red blood cells [46].

### Isolation and culture of DC

Human DC were generated *in vitro *from blood-derived adherent peripheral monocytes as already described [47]. Briefly, human PBMC obtained from venous blood of healthy donors (Blood Bank SRK, Zürich, Switzerland) were isolated by Ficoll-Paque (Pharmacia Biotech, Uppsala, Sweden) density centrifugation. The PBMC were cultured in RPMI 1640 supplemented with penicillin/streptomycin (all Life Technologies, Paisley, UK) and 10% heat-inactivated pooled human A serum (Blood Bank SRK) for 2 h. The adherent cells were cultured for 6 days in DC culture medium composed of RPMI 1640 supplemented with penicillin/streptomycin, 5% heat-inactivated pooled human A serum, rGM-CSF (50 ng/ml, Novartis, Basel, Switzerland), and rIL-4, (100 U/ml, R&D Systems, Abingdon, UK).

### Challenge of DC with Listeria

*L. innocua *were propagated in TSB at 37°C overnight, diluted 10-fold and further expanded to an OD_600 _of 0.5 corresponding to 5 × 10^7^/ml viable bacteria. Bacteria were harvested by centrifugation, washed twice with PBS prior to opsonization in RPMI 1640 with 50% pooled heat-inactivated human A serum for 30 minutes at 37°C. Opsonized *Listeria *were washed in PBS and resuspended in RPMI 1640. DC were challenged for 1 hour with a multiplicity of infection (MOI) of 5. Subsequently, cultures were washed with PBS and incubated for 3 hours in DC culture medium containing 25 μg/ml of gentamycin (Sigma-Aldrich, St. Louis, MO, USA) to kill extracellular *L. innocua*.

### Viability determination of *L. innocua *and DC

Serial dilutions of *L. innocua *or of *L. innocua*-challenged DC lysate were spread on tryptic soy broth (TSB; Difco Laboratories, Detroit, MI, USA) agar plates. Colony forming units (CFU) were determined by counting colonies after overnight culture at 37°C and specific lysis was calculated using the formula [(CFU in buffer control - CFU in test incubation)/CFU in buffer control] × 100.

Alternatively, turbidimetry was used to study specific lysis of bacteria [48]. Serial dilutions of treated *L. innocua *or cell lysates were incubated in 96-well plates (Nunc). Bacterial growth curves were monitored in a microplate reader at OD_600 _while discontinuous shaking for 16 hours at 37°C. Specific lysis was calculated by determining the time when the maximum population was reached in buffer controls (OD_Tmax-control_). At this time point the OD value (OD_Tmax-Test_) of a shifted growth curve was evaluated and specific lysis was calculated using the formula [(OD_Tmax-control _- OD_min_) - (OD_Tmax-Test _- OD_min_)/OD_Tmax-control _- OD_min_] × 100. All OD values were corrected by subtraction of the baseline OD (OD_min_).

Lysis of DC during granulysin and perforin incubation was determined in LDH release assays using the cytotoxicity detection kit (Roche, Basel Switzerland) following the manufacturers instruction. In brief, DC were seeded in a 96-well plate (NUNC) and were challenged with *L. innocua *in a MOI of 10 prior to treatment with various granulysin and/or perforin concentration for indicated times. For cell lysis analysis 100 μl of cell-free supernatant was harvested, mixed with dye solution, incubated for 20 minutes and absorption was measured at 490 nm. Percent specific lysis was calculated according to the formula: ((experimental value - spontaneous release)/(maximum release - spontaneous release) × 100. Spontaneous release corresponded to untreated DC and cells lysed with Triton-X 100 showed the maximal release.

### Bacteriolytic activity of granulysin and perforin

Granulysin in the presence or absence of perforin or perforin alone was incubated at various concentrations for 3 hours at either 4°C or 37°C with *Listeria*-challenged DC or with 10^5^/ml *L. innocua *in 0.01 M Trisma^R ^base (pH 8, Sigma-Adrich). Actin_frag_, medium or buffer served as controls. For some experiments perforin was replaced by ionomycin in a concentration of 1 μM. After incubation, the viability of *L. innocua *was determined as described above. Alternatively, induction of listeriolysis was tested using a pulse-chase experimental system. For this purpose, DC were pulsed with granulysin (2.5 μM) for 15 minutes on ice before the medium was replaced and DC further incubated at 37°C. Perforin (2.5 kU/ml) was added at indicated time points for the remaining experimental duration (max. 180 minutes). In addition, some samples were perforin treated for 15 minutes at 37°C prior to medium exchange and further incubation with granulysin for another 180 minutes.

To quantify granulysin binding and uptake *L. innocua*-challenged DC were incubated with 2.5 μM granulysin and perforin in indicated concentrations for 45 minutes at 37°C or at 4°C, subsequently washed three times for 5 minutes with 10 mM arginine in PBS to remove granulysin bound outside at the cell membrane or PBS alone as control. DC were finally lysed with PBS containing 0.5% Triton-X-100. The content of granulysin bound or internalized in DC was determined by Western blot analysis. Samples were run on a 15% SDS-PAGE gel and blotted onto transfer membranes (Immobilon-P; Millipore). Granulysin was detected using the anti His mAb (1:1000; Invitrogen, Carlsbad, CA, USA). As a reference, cellular actin was detected using an anti-actin mAb (AC15; Sigma-Aldrich). The granulysin content in DC was measured and calculated relative to cellular actin using Image-J software (National Institutes of Health).

### Confocal laser scanning microscopy

*L. innocua*-challenged DC were incubated with various concentrations of granulysin, actin_frag _or culture medium alone in presence or absence of perforin for indicated times either at 37°C or at 4°C, subsequently washed twice with PBS and fixed with 1.5% PFA in PBS containing 1% sucrose for immunofluorescence labelling. Fixed DC were scratched and cytospun onto glass slides and permeabilized with 0.1% Triton X-100 (Sigma-Aldrich) in PBS for 1 minute at room temperature (RT). Unspecific binding was blocked with 0.1% BSA bovine serum albumin (BSA; Fluka, Buchs, Switzerland) in PBS for 1 hour RT. Recombinant His-tagged granulysin was detected with a monoclonal anti-His Ab (1:1000). Early endosomes were detected with a rabbit EEA-1 Ab (1:200; Affinity BioReagents, Golden, CO, USA). Omitting the first Abs served as control for specificity. For detection the following Abs were used: FITC-conjugated goat anti-mouse or a goat anti-rabbit Ab (all KPL Inc., Gaithersburg, Maryland, USA), or Texas Red-conjugated donkey anti-mouse Ab (Jackson Immuno Research Laboratories, West Grove, PA, USA). All Abs were diluted in 0.1% BSA in PBS. DNA was labelled with 1 μg/ml DAPI (4,6-diamidine-2-phenyl-indol-dihydrochlorid, Roche, Mannheim, Germany) in PBS for 15 minutes at RT. Fluorescent labelled specimens were examined using a confocal laser scanning microscope (CLSM SP1, Leica, Heidelberg, Germany).

For the detection of plasma membrane pores *Listeria*-challenged DC were incubated with perforin at the indicated concentration for 45 minutes at 37°C in medium containing 2 μM ethidium homodimer-2 (EthD-2, Invitrogen).

Images were analysed using Imaris software package (Bitplane, Zurich, Switzerland) and threshold levels for calculation of colocalization micrographs (granulysin and EEA-1) were selected above background signals. The degree of colocalization of granulysin with bacterial DNA spots was determined by counting all intracellular distinguishable bacteria as well as those clearly coated by granulysin in at least 200 highly magnified DC per time point.

### Ca^2+ ^measurements

For measuring intracellular Ca^2+^, *Listeria*-challenged or unchallenged DC grown on coverslips were loaded with Fura-2 (Molecular Probes) in a concentration of 10 μM in DC culture medium for 15 minutes at room temperature and were then mounted on a thermostatically controlled chamber maintained at 37°C on an inverted microscope (Zeiss Axiovert 200) equipped with a video-imaging system. For some experiments DC were pretreated with 10 μM dantrolene and 25 μM TMB-8 for 15 minutes at room temperature. Prior to incubation with granulysin and/or perforin in various concentrations and time periods the coverslips were rinsed with incubation buffer (145 mM NaCl, 1.6 mM K_2_HPO_4_, 0.4 mM KH_2_PO4, 5 mM D-Glucose, 1 mM MgCl_2_, 1.3 mM Ca^2+^-Gluconate) until stable imaging was achieved. Some samples were rinsed in low Ca^2+ ^buffer (145 mM NaCl, 1.6 mM K_2_HPO_4_, 0.4 mM KH_2_PO4, 5 mM D-Glucose, 1 mM MgCl_2_, 1.3 μM Ca^2+^-Gluconate). As positive control for the elevation of intracellular Ca^2+ ^served ATP (1 mM) as well as ionomycin (1 μM). The specimens were excited with light of 340/380-nm wavelengths and the excitations ratio 340/380 was monitored. After certain time periods (30–60 seconds) fluorescent images (Ex 340 nm; Em 525 nm) were recorded.

## Authors' contributions

MW participated in the conception of the study, in the performance of the experiments, in the analysis of the data and drafted the manuscript. SLG participated in the infections, viability testing, morphology experiments and data analysis. AV and CAW participated in the Ca^2+ ^measurements and interpretation of the data. HS and CD participated in the analysis and interpretation of the data.

PG provided critical intellectual input to the study and organized financial support. UZ participated in the conception and coordination of the study, contributed to the interpretation of the data and helped to draft the manuscript. All authors read and approved the final manuscript.

## References

[B1] Lara-Tejero M, Pamer EG (2004). T cell responses to Listeria monocytogenes. Curr Opin Microbiol.

[B2] Stenger S (2001). Cytolytic T cells in the immune response to mycobacterium tuberculosis. Scand J Infect Dis.

[B3] Flynn JL, Chan J, Triebold KJ, Dalton DK, Stewart TA, Bloom BR (1993). An essential role for interferon gamma in resistance to Mycobacterium tuberculosis infection. J Exp Med.

[B4] Kaufmann SH (1999). Cell-mediated immunity: dealing a direct blow to pathogens. Curr Biol.

[B5] Gansert JL, Kiessler V, Engele M, Wittke F, Rollinghoff M, Krensky AM, Porcelli SA, Modlin RL, Stenger S (2003). Human NKT cells express granulysin and exhibit antimycobacterial activity. J Immunol.

[B6] Manning WC, O'Farrell S, Goralski TJ, Krensky AM (1992). Genomic structure and alternative splicing of 519, a gene expressed late after T cell activation. J Immunol.

[B7] Jongstra J, Schall TJ, Dyer BJ, Clayberger C, Jorgensen J, Davis MM, Krensky AM (1987). The isolation and sequence of a novel gene from a human functional T cell line. Journal of Experimental Medicine.

[B8] Stenger S, Hanson DA, Teitelbaum R, Dewan P, Niazi KR, Froelich CJ, Ganz T, Thoma-Uszynski S, Melian A, Bogdan C (1998). An antimicrobial activity of cytolytic T cells mediated by granulysin. Science.

[B9] Walch M, Eppler E, Dumrese C, Barman H, Groscurth P, Ziegler U (2005). Uptake of granulysin via lipid rafts leads to lysis of intracellular Listeria innocua. J Immunol.

[B10] Young JD, Hengartner H, Podack ER, Cohn ZA (1986). Purification and characterization of a cytolytic pore-forming protein from granules of cloned lymphocytes with natural killer activity. Cell.

[B11] Podack ER, Young JD, Cohn ZA (1985). Isolation and biochemical and functional characterization of perforin 1 from cytolytic T-cell granules. PNAS.

[B12] Young JD, Cohn ZA, Podack ER (1986). The ninth component of complement and the pore-forming protein (perforin 1) from cytotoxic T cells: structural, immunological, and functional similarities. Science.

[B13] Trapani JA, Smyth MJ (1993). Killing by cytotoxic T cells and natural killer cells: multiple granule serine proteases as initiators of DNA fragmentation. Immunol Cell Biol.

[B14] Froelich CJ, Orth K, Turbov J, Seth P, Gottlieb R, Babior B, Shah GM, Bleackley RC, Dixit VM, Hanna W (1996). New paradigm for lymphocyte granule-mediated cytotoxicity. Target cells bind and internalize granzyme B, but an endosomolytic agent is necessary for cytosolic delivery and subsequent apoptosis. J Biol Chem.

[B15] Metkar SS, Wang B, Aguilar-Santelises M, Raja SM, Uhlin-Hansen L, Podack E, Trapani JA, Froelich CJ (2002). Cytotoxic cell granule-mediated apoptosis: perforin delivers granzyme B-serglycin complexes into target cells without plasma membrane pore formation. Immunity.

[B16] Keefe D, Shi L, Feske S, Massol R, Navarro F, Kirchhausen T, Lieberman J (2005). Perforin Triggers a Plasma Membrane-Repair Response that Facilitates CTL Induction of Apoptosis. Immunity.

[B17] Canaday DH, Wilkinson RJ, Li Q, Harding CV, Silver RF, Boom WH (2001). CD4(+) and CD8(+) T cells kill intracellular Mycobacterium tuberculosis by a perforin and Fas/Fas ligand-independent mechanism. J Immunol.

[B18] Ochoa MT, Stenger S, Sieling PA, Thoma-Uszynski S, Sabet S, Cho S, Krensky AM, Rollinghoff M, Nunes Sarno E, Burdick AE (2001). T-cell release of granulysin contributes to host defense in leprosy. Nat Med.

[B19] Hof H, Hefner P (1988). Pathogenicity of Listeria monocytogenes in comparison to other Listeria species. Infection.

[B20] Podack ER, Lowrey DM, Lichtenheld M, Hameed A (1988). Function of granule perforin and esterases in T cell-mediated reactions. Components required for delivery of molecules to target cells. Ann N Y Acad Sci.

[B21] Salter MW, Hicks JL (1995). ATP causes release of intracellular Ca2+ via the phospholipase C beta/IP3 pathway in astrocytes from the dorsal spinal cord. J Neurosci.

[B22] Nishizaki T (2004). ATP- and adenosine-mediated signaling in the central nervous system: adenosine stimulates glutamate release from astrocytes via A2a adenosine receptors. J Pharmacol Sci.

[B23] Remy C, Kirchhoff P, Hafner P, Busque SM, Mueller MK, Geibel JP, Wagner CA (2007). Stimulatory pathways of the Calcium-sensing receptor on acid secretion in freshly isolated human gastric glands. Cell Physiol Biochem.

[B24] O'Connell PJ, Klyachko VA, Ahern GP (2002). Identification of functional type 1 ryanodine receptors in mouse dendritic cells. FEBS Lett.

[B25] Stolk M, Leon-Ponte M, Merrill M, Ahern GP, O'Connell PJ (2006). IP3Rs are sufficient for dendritic cell Ca2+ signaling in the absence of RyR1. J Leukoc Biol.

[B26] Shi L, Keefe D, Durand E, Feng H, Zhang D, Lieberman J (2005). Granzyme B binds to target cells mostly by charge and must be added at the same time as perforin to trigger apoptosis. J Immunol.

[B27] Pinkoski MJ, Hobman M, Heibein JA, Tomaselli K, Li F, Seth P, Froelich CJ, Bleackley RC (1998). Entry and trafficking of granzyme B in target cells during granzyme B-perforin-mediated apoptosis. Blood.

[B28] Pieters J (2001). Entry and survival of pathogenic mycobacteria in macrophages. Microbes Infect.

[B29] Walburger A, Koul A, Ferrari G, Nguyen L, Prescianotto-Baschong C, Huygen K, Klebl B, Thompson C, Bacher G, Pieters J (2004). Protein kinase G from pathogenic mycobacteria promotes survival within macrophages. Science.

[B30] Trapani JA (1995). Target cell apoptosis induced by cytotoxic T cells and natural killer cells involves synergy between the pore-forming protein, perforin, and the serine protease, granzyme B. Aust N Z J Med.

[B31] Trapani JA, Sutton VR, Thia KY, Li YQ, Froelich CJ, Jans DA, Sandrin MS, Browne KA (2003). A clathrin/dynamin- and mannose-6-phosphate receptor-independent pathway for granzyme B-induced cell death. [see comment]. J Cell Biol.

[B32] Okada S, Li Q, Whitin JC, Clayberger C, Krensky AM (2003). Intracellular mediators of granulysin-induced cell death. J Immunol.

[B33] Burster T, Giffon T, Dahl ME, Bjorck P, Bogyo M, Weber E, Mahmood K, Lewis DB, Mellins ED (2007). Influenza A virus elevates active cathepsin B in primary murine DC. Int Immunol.

[B34] Balaji KN, Schaschke N, Machleidt W, Catalfamo M, Henkart PA (2002). Surface cathepsin B protects cytotoxic lymphocytes from self-destruction after degranulation. J Exp Med.

[B35] Baran K, Ciccone A, Peters C, Yagita H, Bird PI, Villadangos JA, Trapani JA (2006). Cytotoxic T lymphocytes from cathepsin B-deficient mice survive normally in vitro and in vivo after encountering and killing target cells. J Biol Chem.

[B36] McNeil PL, Steinhardt RA (2003). Plasma membrane disruption: repair, prevention, adaptation. Annu Rev Cell Dev Biol.

[B37] Steinhardt RA, Bi G, Alderton JM (1994). Cell membrane resealing by a vesicular mechanism similar to neurotransmitter release. Science.

[B38] Terasaki M, Miyake K, McNeil PL (1997). Large plasma membrane disruptions are rapidly resealed by Ca2+-dependent vesicle-vesicle fusion events. J Cell Biol.

[B39] Togo T, Alderton JM, Steinhardt RA (2003). Long-term potentiation of exocytosis and cell membrane repair in fibroblasts. Mol Biol Cell.

[B40] Malik ZA, Thompson CR, Hashimi S, Porter B, Iyer SS, Kusner DJ (2003). Cutting edge: Mycobacterium tuberculosis blocks Ca2+ signaling and phagosome maturation in human macrophages via specific inhibition of sphingosine kinase. J Immunol.

[B41] Jahraus A, Tjelle TE, Berg T, Habermann A, Storrie B, Ullrich O, Griffiths G (1998). In vitro fusion of phagosomes with different endocytic organelles from J774 macrophages. J Biol Chem.

[B42] Gorvel JP, Chavrier P, Zerial M, Gruenberg J (1991). rab5 controls early endosome fusion in vitro. Cell.

[B43] Lawe DC, Sitouah N, Hayes S, Chawla A, Virbasius JV, Tuft R, Fogarty K, Lifshitz L, Lambright D, Corvera S (2003). Essential role of Ca2+/calmodulin in Early Endosome Antigen-1 localization. Mol Biol Cell.

[B44] Malik ZA, Denning GM, Kusner DJ (2000). Inhibition of Ca(2+) signaling by Mycobacterium tuberculosis is associated with reduced phagosome-lysosome fusion and increased survival within human macrophages. J Exp Med.

[B45] Froelich CJ, Turbov J, Hanna W (1996). Human perforin: rapid enrichment by immobilized metal affinity chromatography (IMAC) for whole cell cytotoxicity assays. Biochem Biophys Res Commun.

[B46] Henkart PA, Yue CC, Yang J, Rosenberg SA (1986). Cytolytic and biochemical properties of cytoplasmic granules of murine lymphokine-activated killer cells. J Immunol.

[B47] Filgueira L, Nestle FO, Rittig M, Joller HI, Groscurth P (1996). Human dendritic cells phagocytose and process Borrelia burgdorferi. J Immunol.

[B48] Begot C, Desnier I, Daudin JD, Labadie JC, Lebert A (1996). Recommendations for calculating growth parameters by optical density measurements. J Microbiol Meth.

